# Processing negative autobiographical memories in a foreign language

**DOI:** 10.3389/fpsyg.2023.1133915

**Published:** 2023-05-16

**Authors:** Isabel Ortigosa-Beltrán, Irene Jaén, Azucena García-Palacios

**Affiliations:** ^1^Center for Brain and Cognition, Universitat Pompeu Fabra, Barcelona, Spain; ^2^Department of Basic and Clinical Psychology and Psychobiology, Jaume I University, Castellón, Spain

**Keywords:** foreign language, bilingualism, autobiographical memories, moderation, emotion

## Abstract

The use of a foreign language has been introduced in the clinical setting as a form of emotional distance to help deal with negative experiences. However, the evidence of foreign language reducing emotionality during processing negative events is still scarce. This study aims to test whether the description and processing of a traumatic or highly emotional event in a foreign language could modulate the strength of the connection between traumatic symptomatology and emotional reaction. For this purpose, a sample of 128 healthy participants completed a series of questionnaires *via* an online platform. Firstly, their levels of distress, arousal and valence were assessed in their native language. Secondly, they were assigned to either the native language or the foreign language group and described a negative childhood event in the assigned language (English or Spanish), followed by five questions for processing the event. Next, their emotionality was assessed again in their native language. Finally, a questionnaire of traumatic stress symptoms and an avoidance scale were completed. Results showed that the relationship between traumatic symptomatology and emotionality was moderated by the language of processing the negative event. Specifically, traumatic symptomatology was more strongly associated with distress and arousal change when the processing task was performed in the native language. These findings suggest the influence of a foreign language on emotional reactivity when a negative experience is processed, which could be an essential tool in the treatment of disorders related to stress and trauma.

## 1. Introduction

Traumatic symptoms are narrowly associated with an increase in negative emotional intensity (Amstadter and Vernon, 2008), which have been directly related to emotional brain regions such as the amygdala and the hippocampus, as well as areas involving intense negative memories (Jacques et al., 2011). Repressing and storing feelings associated with unpleasant or painful memories has been a key topic in psychological tradition. Unravelling and communicating the distressing information we keep to ourselves has long been considered a healthy habit (Frattaroli, 2006), and has been associated with improvements in mental health and recovery (Bistrović et al., 2013). As a method to draw and shape painful past events, emotional disclosure is commonly achieved through a linguistic avenue, either written or verbalised (Pennebaker, 1993; Lepore and Smyth, 2002). However, it is a process that may become resistant due to the patient’s avoidance related to trauma. Also, patients can find the treatment painful, making it difficult to deal with the negative experience. These resistances have resulted in a growing interest in looking for appropriate psychotherapeutic responses to improve the treatment experience (e.g., Chu, 1988; Bicknell-Hentges and Lynch, 2009).

Some authors suggest that emotions can be influenced by aspects such as the use of a native or a foreign language, understanding the latter as that acquired in an educational setting at a later stage in life (Caldwell-Harris, 2014). Specifically, using a foreign language works as an intermediary or buffer that softens the impact of the emotional distress inherent to strong affective information (Iacozza et al., 2017). In this regard, a study conducted by García-Palacios et al. (2018) showed that conditioning of fear was lighter in a foreign language in comparison to the native one suggesting that the foreign language context can help to reduce emotional reactivity and take distance from the situation. Also, Ortigosa Beltrán et al. (in review) explored the effect of a foreign language during cognitive reappraisal. They showed that using a foreign language could be advantageous in reducing negative emotionality produced by phobic stimuli through this emotion regulation strategy. In addition, in an experiment on cross-language processing of emotional texts, Dylman and Bjärtå (2018) showed that reading and answering questions regarding negative text extracts in a foreign language was associated with lower distress than using the native language. In the same line, Anooshian and Hertel (1994) found a better recall of emotional words in late bilinguals in the native language in comparison to the foreign language, suggesting as a possible interpretation that participants were paying more attention to the pronunciation in the latter. Furthermore, in an event-related potential study, Jończyk et al. (2016) showed a stronger emotional response when participants read negative sentences in their native language compared with a foreign language.

Based on these studies, using a foreign language seems to be associated with decreased emotional reactivity in emotional tasks compared to using the native language, which is often explained by an increased psychological distance (Shin and Kim, 2017). In this regard, a review conducted by Pavlenko (2012) suggested that using a foreign language decreased the automaticity of affective processing, reducing interference effects and decreasing electrodermal activity reactivity to negative emotional stimuli. In addition, research on a neurobiological level has indeed identified some mechanisms revealing differences in the use of cognitive resources between the use of native and foreign languages (see Perani and Abutalebi, 2005). Abutalebi (2008) points out that the grammatical and lexico-semantic processing in a foreign language demands more cognitive resources, as shown by the recruitment of cognitive control brain areas, Also, Branzi et al. (2016) showed that using a foreign language leads to greater recruitment of neural areas involved with cognitive control, which could be associated with a detaching effect. In fact, research has shown that cognitive resources when using a foreign language may be modulated by the proficiency level or the age of acquisition of the foreign language. Specifically, the recruitment of cognitive resources was greater when the foreign language was less proficient or the age of acquisition was in a later stage (Perani and Abutalebi, 2005), factors that also have been associated with decreased affective processing (Pavlenko, 2012). As some research has suggested, greater task difficulty is associated with lower emotionality (Jasinska et al., 2012). Therefore, using a language a foreign language, which implies a greater difficulty than using the native one, could be associated with a lower impact of emotion.

Nevertheless, other studies have not shown this pattern of emotional distance when a foreign language is used. Specifically, Ortigosa-Beltrán et al. (2023) showed no differences between native and foreign languages in fear extinction during a laboratory task. Specifically, the study suggested that using a foreign language may reduce the emotional impact of a stimulus during the process of conditioning. However, the process of extinction could be not a suitable context to observe the foreign language effect since using a foreign language may not necessarily result in increased safety responses. Safety cues already imply a reduction in emotional intensity, and it is possible that the foreign language effect did not have an additive effect in reducing emotional intensity in this context. Also, Ferré et al. (2010) showed no differences between using the native or the foreign language to recall emotional words. In addition, this study did not find any effect of dominance and the context or the age of language acquisition. Other authors have shown equivalent results in different tasks, such as similar reaction times to negative words (Grabovac and Pléh, 2014) or the Affective Simon Task (Altarriba and Basnight-Brown, 2011). These divergences highlight the need to test the effect of using a foreign language using distinct psychological processes.

In the context of clinical settings, Marcos and his group (Marcos and Alpert, 1976; Marcos, 1988) explored the dynamic of bilinguals in the psychotherapeutic context through a series of clinical cases and empirical studies exploring how bilingual persons manifest certain aspects of themselves depending on the language used. They suggested that language switching in the psychotherapy process may have inhibitory or facilitatory effects. Bilinguals can use their foreign language, which they refer is a more detached abstract language, to avoid anxiety. However, using a foreign language can also be useful when it is used constructively to approach conflicting material. Thus, switching may be used strategically for the therapist according to specific diagnostic features of the patient. Also, Pitta et al. (1978) also emphasised the use of switching languages by the psychotherapist as an effective strategy to facilitate the efforts to objectify emotionally charged issues for their hysterical clients.

The foreign language effect has received special attention in the area of recalling traumatic memories, which are especially painful to retrieve. Autobiographical memories are composed of sensory information in the form of visual images, spatial or auditory imagery, and a certain degree of emotional charge and language (Greenberg and Rubin, 2003). There is no doubt that language plays a primordial role in the processing of autobiographical memories, making possible the encoding and expression of these memories (Donald, 2012). Studies reported that bilingual people who retrieve their memories using a foreign language expressed their experiences in a more distant (Aragno and Schlachet, 1996) and elaborated way (Javier et al., 1993; Schrauf, 2000), compared with those recalling their experience in their native language, the language of the memory acquisition. In this regard, Schwanberg (2010) showed that matching the language at the time that trauma has been experienced and the language of retrieval facilitates the healing process by allowing unique access to the traumatic memories through the first language. In addition, Jansson and Dylman (2021) found that using a foreign language modulated the ratings on emotionality, vividness and intrusive memories after reactivating emotional autobiographical memories, compared to a native language. Although the use of the individual’s native language may be preferred for accessing traumatic memories during the healing process, psychotherapy can also use the foreign language as a tool to retrieve negative memories and help in dealing with those traumatic events that generate discomfort (Larsen et al., 2002). However, it is unknown to what extent using a different language may modulate the relationship between traumatic symptoms related to a negative memory and the emotional response when describing and processing that event.

In essence, language constitutes a contextual component that can influence the way in which we access and retrieve a memory. The foreign language effect in the recall of traumatic memories needs deeper exploration since it may contribute to a better approach in therapy. Patients are commonly reluctant to retrieve emotional events due to the high anxiety and stress levels when remembering them, leading to the avoidance of thinking or talking about the memory (Foa et al., 2002). In this sense, the foreign language effect could function as a tool influencing how patients process and perceive their emotions. The current study goes in this direction, examining the influence of language on the relationship between post-traumatic symptomatology and emotional reactivity in terms of rating of distress, arousal and emotional valence. We hypothesise that a positive relationship between post-traumatic symptomatology and emotional reactivity (distress, arousal and emotional valence) will be found, and this relationship will be stronger when the negative memory is processed in the native language, compared with a foreign language.

## 2. Methods

### 2.1. Participants

The sample size was calculated by G*Power (Faul et al., 2009), revealing a sample of 119 participants, with an effect size of 0.15 and a power of 0.95 for regression analysis with 3 predictors (post-traumatic stress symptoms, language, and interaction). A total of 128 participants (60 women; 63 men; 5 other gender; mean age = 28.70; SD = 9.02) took part in the online questionnaire. All were healthy population and gave informed consent to participate in the study. The inclusion criteria were (1) using Spanish as their mother tongue and (2) a high degree of English proficiency in terms of their self-reported knowledge, fluency, and use of the language (see Table 1). Differences in terms of English proficiency between participants who performed the task in their native language or foreign language were not observed (mean native group = 7.84; SD = 1.2; mean foreign group = 7.56; SD = 1.2). The most frequent ranges of age of foreign language acquisition were between 5 and 10 years old (64%) and before 5 years old (17.2%). A total of 13 participants were excluded as they rated the event with a score lower than 5 (out of 10) on the scale of painfulness, or they reported not remembering any negative event of their childhood, resulting in a final sample of 115 participants.

**Table 1:** Participants´ basic language skills in the native and in foreign language groups (means and standard deviations).

	Native (*n* = 64)	Foreign (*n* = 64)
General	7.84 (1.26)	7.56 (1.23)
Speaking	7.20 (1.52)	6.90 (1.50)
Listening	8.20 (1.22)	7.58 (1.45)
Writing	8.03 (1.44)	7.60 (1.40)
Reading	8.73 (0.98)	8.40 (1.25)

### 2.2. Measures

#### 2.2.1. English skills

An adaptation from the LEAP-Q (Marian et al., 2007) was used to assess self-reported of knowledge of English skills. The dimensions assessed were writing, speaking, listening and reading. All items were presented on a scale from 1 to 10. This questionnaire was included in the study to control the eligibility and study plausible individual linguistic differences between participants who respond in their native language and those who respond in a foreign language. Additionally, participants also reported having English as a foreign language as one of their characteristics within the platform Prolific.

#### 2.2.2. Post-traumatic stress symptomatology

The Davidson Trauma Scale (DTS; Davidson et al., 1997) is a 17-item questionnaire to self-report the frequency and severity of post-traumatic stress disorder symptoms in the past week. Scores range from “0 = not at all” to “4 = every day” for frequency and from “0 = not at all distressing” to “4 = extremely distressing” for severity. In this study, participants were asked to answer the questions of this scale focusing on the experience recalled in relation to a negative event. For this study, the DTS’s internal consistency was adequate (Cronbach alfa = 0.97).

#### 2.2.3. Distress

The subjective Units of Distress Scale (SUDS; Wolpe, 1969) was used to measure distress and discomfort, following Dylman and Bjärtå (2018). This scale ranges from 0 (no distress) to 10 (high distress). Previous experiments used it as a scale of distress and anxiety (Tanner, 2012).

#### 2.2.4. Valence and arousal

The Self-Assessment Manikin (SAM; Lang, 1980) was administered to assess ratings of valence of their emotions and the self-report of arousal (people’s reports of affective experience) as a non-verbal scale. The scale of valence ranged between 1 (positive valence) and 5 (negative valence), and the scale of arousal also ranged between 1 (high arousal) and 5 (low arousal).

#### 2.2.5. Processing questions protocol

The participants were instructed to describe a negative, painful or traumatic childhood event in as detail as possible. Next, they rated their levels of painfulness and interference related to this event on a scale from 0 to 10. The event description was considered part of the processing in this study since recalling an autobiographical memory could increase the negative effect (Slofstra et al., 2017). These events were classified and shown in Table 2. Afterwards, they completed five processing questions adapted from the protocol by Foa and Rothbaum (1998) for post-traumatic stress disorder (PTDS). Specifically, these questions were: (1) which clothes were they wearing at this moment? (2) who else was around? (3) what went through your mind in that situation? (4) Have you discovered any capabilities or strengths that you were unaware of after the event occurred? (5) How do you think you will see the event when you are 20 or 30 years older? What meaning will it have for you? Finally, participants were asked about how much they did want to avoid the situation (0 = I did not want to stop remembering the event; 10 = I really want to stop remembering the event).

**Table 2:** Levels of painfulness and interference according to the category of the events described in each language (percentage of the frequency of the event, means and standard deviations).

Category of the events	Native language				Foreign language			
	%	Painfulness *M* (SD)	Interference *M* (SD)	Avoidance *M* (SD)	%	Painfulness *M* (SD)	Interference *M* (SD)	Avoidance *M* (SD)
Episode of physical or verbal aggression by other children	23.4	7.87 (1.40)	3.67 (2.94)	46.87 (30.06)	23.4	7.07 (2.18)	4.20 (0.67)	53.33 (38.47)
Family incident	29.7	7.63 (1.73)	3.95 (2.85)	43.58 (35.92)	34.4	7.55 (1.56)	3.68 (0.94)	40.09 (34.85)
School incident	0	0	0	0	14.1	6.11 (2.84)	4.11 (0.92)	35.67 (35.31)
Accident	10.9	6.43 (2.14)	2.71 (3.30)	30.00 (23.20)	6.3	7.00 (2.16)	3.25 (0.95)	40.00 (34.76)
Own illness	9.4	8.50 (1.04)	2.17 (2.48)	27.17 (37.33)	3.1	8.50 (0.70)	4.50 (0.70)	65.50 (21.92)
Illness of a family member	3.1	9.00 (1.41)	1.50 (2.12)	75.50 (20.50)	0	0	0	0
Sexual abuse	0	0	0	0	3.1	9.00 (1.41)	4.00 (0)	73.50 (37.47)
Death of a close one	20.3	7.92 (2.59)	2.15 (2.34)	34.77 (36.00)	14.1	8.33 (2.23)	4.56 (0.52)	62.67 (35.5)
No memory	3.1	–	–	–	1.6	–	–	–

### 2.3. Design and procedure

**Figure 1:**
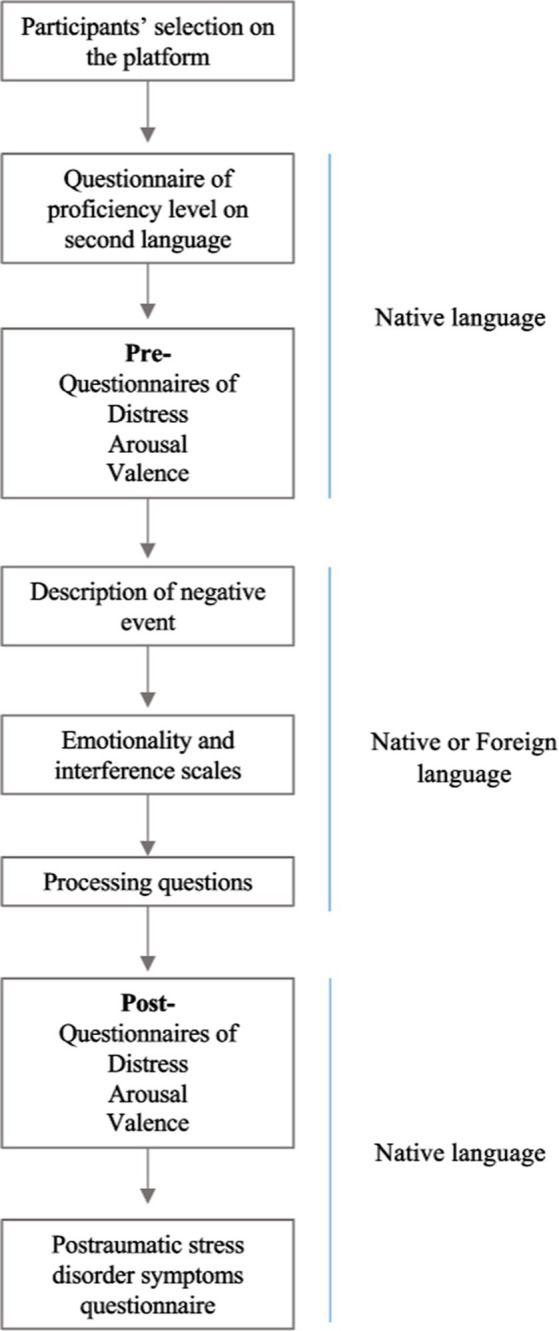
Procedure flowchart.

This is a cross-sectional study in which participants completed an online survey displayed with the online platform Qualtrics^1^ and disseminated by Prolific.^2^ The study was approved by Universitat Jaume I ethical committee (CD/100/2021). Participants entered the platform according to the criteria related to the native language (Spanish), country of birth and residence (Spain) and a general estimate of the level of English (good/high level). When the eligibility was confirmed, they were informed of the consent form and asked to provide their email, age, and gender (see Figure 1). All information and questions prior the experiment were provided in the native language, including information on how to complete the task. Then, participants completed the English questionnaire, adapted from the LEAP-Q (Marian et al., 2007), to measure the specific self-perceived level in each English dimension (speaking, listening, writing and reading). Next, they reported the previous levels of distress and emotional affection (valence and arousal) to establish baseline levels. After that, half of the participants proceeded in their native language (Spanish) and the other half in their foreign language (English). They were requested to think briefly and describe a negative event of the past in the corresponding language, following the memory recall paradigm of free recall used previously (Slofstra et al., 2017). Specifically, they were asked to describe a painful, negative or traumatic childhood event following previous literature so that all of them referred to the same period of their lives. Specifically, the exact instruction they received was the following: “Think of an experience from your childhood that was painful, negative or traumatic. Describe this negative event in (corresponding language), do not worry about possible mistakes in the grammar or vocabulary.” Then, participants had to rate the painfulness of the event on a scale from 0 to 10 and the level of interference at present. Next, the five processing questions related to the event were shown in the corresponding language. In the last phase, all participants returned to the native language and completed the same questionnaires as the first phase to determine the level of distress, valence and arousal. They also rate how much they wanted to avoid the situation. Finally, participants completed DTS at the end of the task to not affect the emotional intensity during the processing task. A flowchart of the procedure is shown in Figure 1. Participants took ~10–12 min to complete the entire task. The payment was made through the platform and adjusted to the time spent by each participant (9 euros per hour).

### 2.4. Data analysis

Three *t*-student tests were performed with pre and post-scores of all the dependent variables (distress, valence and arousal) to check the effectivity of the processing task increasing emotionality. Once changes in emotionality were checked, differences between these pre and post-processing scores were calculated to obtain different measures of change in emotionality. Differences were calculated by subtracting the pre-processing value from the post-processing value. Thus, higher change values on SUDS and valence mean more emotional intensity and negative hedonic valence in terms of distress and displeasure, whilst lower change values on arousal indicate more emotional intensity. Also, a one-way ANOVA was conducted to test whether there were differences in post-traumatic stress symptoms between the Native and Foreign groups.

Next, bivariate correlations were analysed to study the association between the study variables (post-traumatic stress symptomatology, distress change, arousal change, valence change and Language). Because language is a categorical variable, correlations with language were measured with the Eta^2^ instead of bivariate correlations, which is an adequate statistic for analysing non-linear relationships (Shaldehi, 2013).

To test the moderating effect of Language on the relationship between post-traumatic stress and emotionality, multiple linear regressions were used *via* the macro PROCESS (Hayes, 2017). Specifically, three regressions were performed, one with each dependent variable (distress, arousal and valence), post-traumatic stress symptomatology as the independent variable, and language as the moderator. Conditional effects of the independent variables on the dependent variables were obtained, as well as a graphical representation to interpret the findings. An alpha level of 0.01 was set for all analyses to reduce Type I errors.

## 3. Results

### 3.1. Characteristics of the negative events

Negative events reported by participants were peer-reviewed and classified into 8 categories (Table 2): (1) episode of physical or verbal aggression by other children, (2) family incidents (e.g., family disputes, family fights, negligence episodes or life changes such as parents’ divorce), (3) School incidents (e.g., anxiety episodes by school competition, problems with teachers), (4) accident, (5) own illness, (6) illness of a family member, (7) sexual abuse, and (8) death of a close one. Overall, the events were rated with a 7.97 for painfulness, 3.69 for interference and 4.72 for avoidance. Three participants reported not remembering any painful, negative or traumatic childhood event and were excluded from subsequent analyses.

Post-traumatic stress symptoms between linguistic groups relating these events were checked, showing no significant differences (Native group: Mean (SD) = 25.83 (26.83); Foreign group: Mean (SD) = 26.50 (23.23); *F* = 0.06; *p* = 0.81).

### 3.2. Emotional reactivity during the processing task

*T*-test showed that the processing task was effective in inducing emotional reactivity, showing higher arousal (Mean_pre_ (SD) = 4.01 (0.84); Mean_post_ (SD) = 3.65 (0.86); *t* = 4.80; *p* < 0.0001; *d* = 0.42), higher distress (Mean_pre_ (SD) *=* 2.37 (1.82); Mean_post_ (SD) *=* 2.92 (1.83); *t* = 4.89; *p* < 0.0001; *d* = 0.30), and lower valence (Mean_pre_ (SD) *=* 2.33 (0.82); Mean_post_ (SD) *=* 2.95 (0.99); *t* = 8.15, *p* < 0.0001; *d* = 0.68) during the post-processing compared with the pre-processing phase. Note that, in this study, lower values indicated higher arousal and valence.

### 3.3. Associations between study variables

Pearson bivariate correlations showed that post-traumatic stress symptoms were positively associated with changes in both distress and valence (distress: *r* = 0.27, *p* = 0.003; valence: *r* = 0.24, *p* = 0.009), this is, with greater distress and displeasure. Also, post-traumatic stress symptoms were negatively associated with the change in arousal values (*r* = −0.23, *p* = 0.012), which indicated higher emotional intensity. Language was not associated with any of the other study variables (Table 3).

**Table 3:** Bivariate correlations between language, post-traumatic stress symptomatology, and changes in distress, valence, and arousal.

	Mean (SD)	Skewness	Kurtosis	Bivariate Pearson correlations and Eta^2^			
				2	3	4	5
1 Language	–			0.0004	0.005	0.006	0.006
2 Post-traumatic stress symptoms	10.70 (4.62)	1.25	1.33		0.27*	0.24*	−0.23*
3 Changes in distress	6.63 (3.45)	1.52	5.45			0.43*	−0.67*
4 Changes in valence	13.81 (6.69)	0.71	2.35				−0.43*
5 Changes in arousal	8.55 (4.30)	−0.85	1.62				

* *p* < 0.001.

### 3.4. Moderation of language

**Figure 2:**
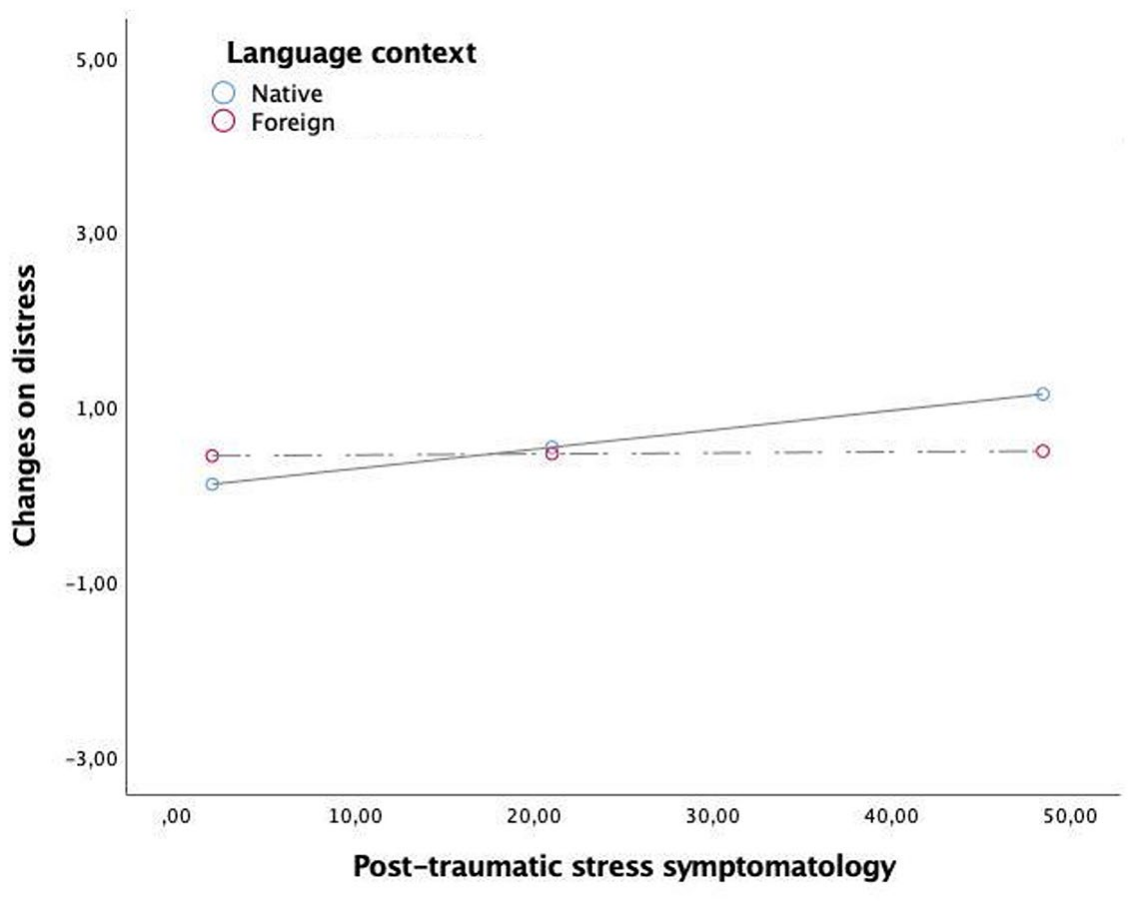
The moderation effect of language context in the relationship between post-traumatic symtomathology and changes on distress.

**Figure 3:**
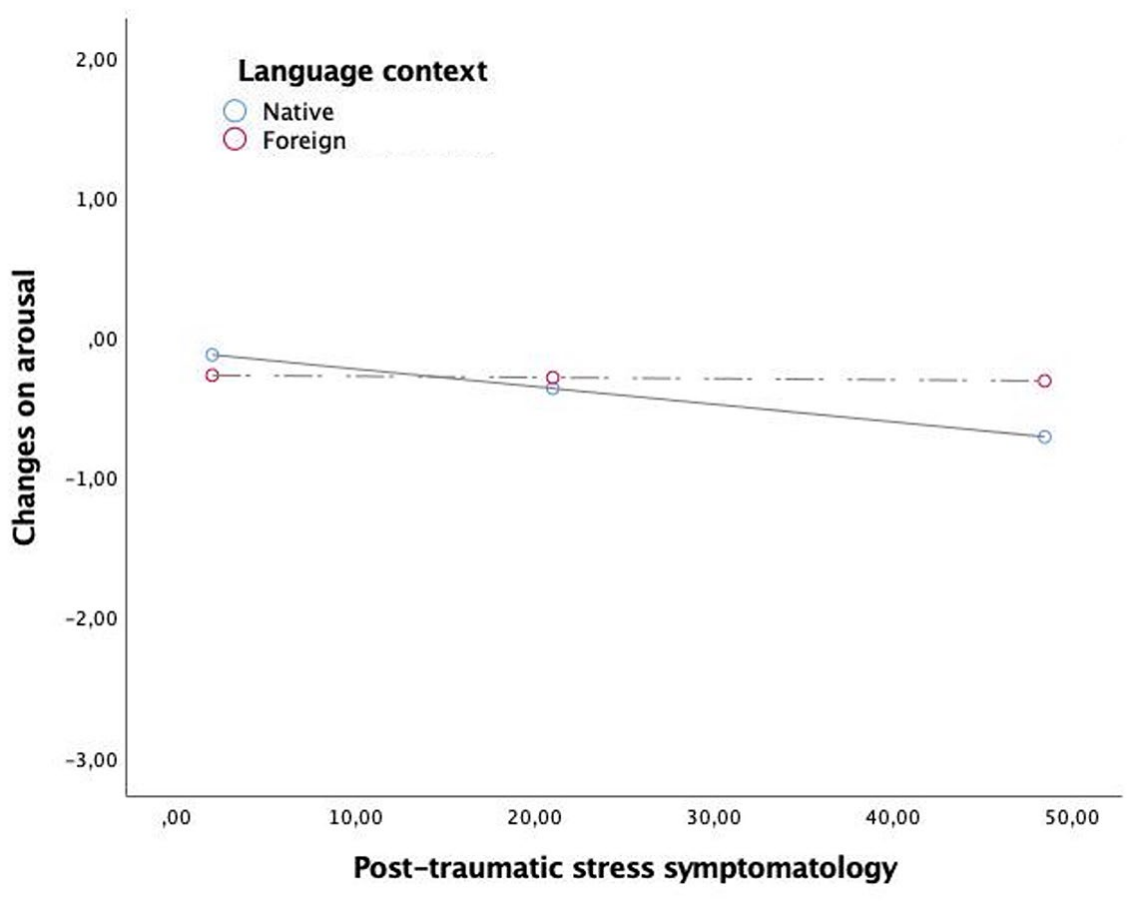
The moderation effect of language context in the relationship between post-traumatic symtomathology and changes on arousal.

**Figure 4:**
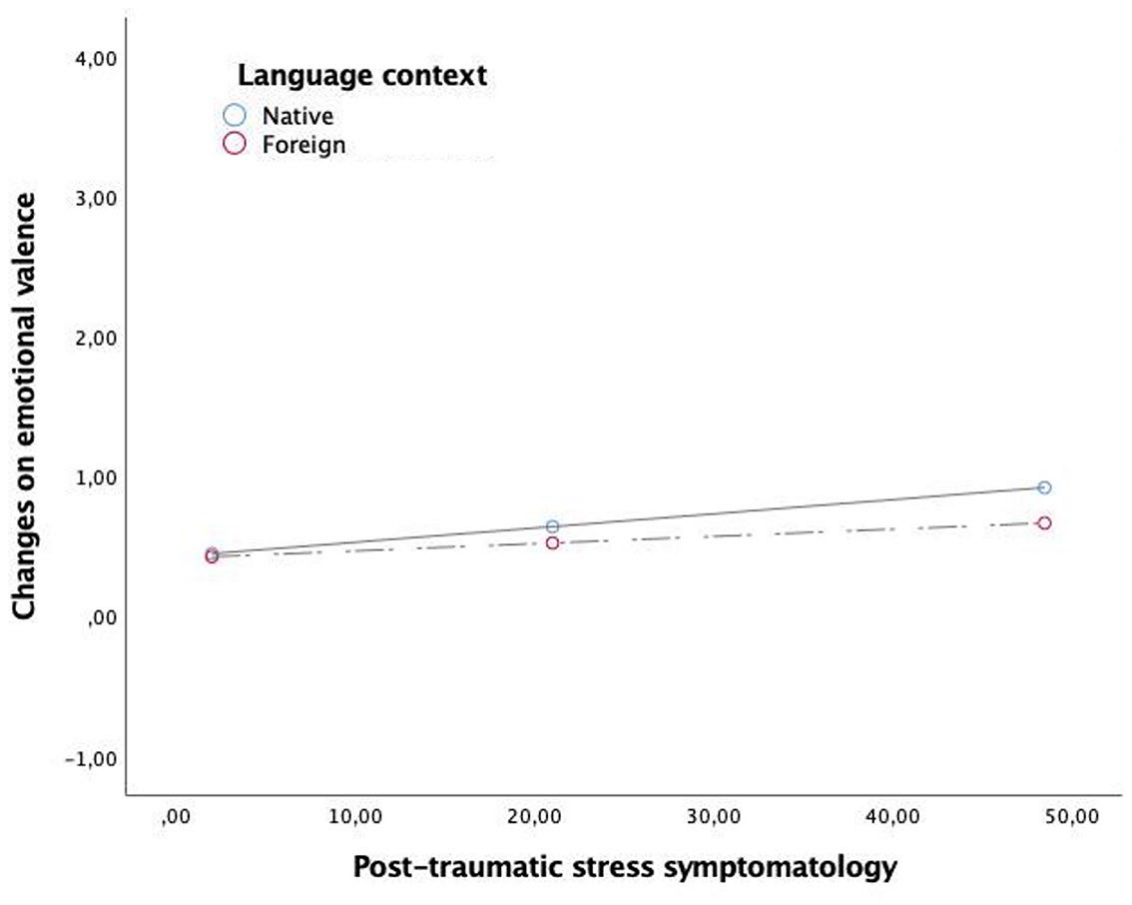
The moderation effect of language context in the relationship between post-traumatic symtomathology and changes on emotional valence.

Results from the multivariate regression analysis are shown in the Table 4. The main effect of post-traumatic stress symptoms was significant for distress (*β* = 0.02, *t* = −3.92 *p* = 0.0002, 99% CI = [0.01, 0.03]), arousal (*β* = −0.01, *t* = −3.29, *p* = 0.0013, 99% CI = [0.63, 0.01]) and valence (*β* = −0.01, *t* = 2.57, *p* = 0.0115, 99% CI = [0.002, 0.018]). Language main effect was not significant for any model. Regarding the moderation effects, results showed that the interaction post-traumatic stress symptomatology x language was significant in fostering distress (*β* = −0.02, *t* = −2.45, *p* = 0.016, 99% CI = [−0.038, −0.040]) and arousal (*β* = 0.01, *t* = 2.02, *p* = 0.045, 99% CI = [0.002, 0.023]), whilst was not significant for valence. As shown in Table 5, the analyses of conditional effects showed that the strength of the relationship between post-traumatic stress symptomatology and emotional reactivity (distress and arousal) was stronger in participants who do the emotional processing in their native language, whilst this relationship decreased being nonsignificant when participants recall and process this memory in a foreign language. This is, the relationship between traumatic symptomatology and distress and arousal was blunted when participants performed the processing task in a foreign language. Graphical representations of the relationship between post-traumatic stress symptomatology and distress or arousal or valence depending on the processing language are shown in Figures 2–4, respectively.

**Table 4:** Multivariate hierarchical regression analyses predicting distress, valence and arousal changes from post-traumatic stress symptoms, language, and their interaction.

	Distress					Valence					Arousal				
	*β*	*t*	*p*-value	99% CI	*R* ^2^	*β*	*t*	*p*-value	99% CI	*R* ^2^	*β*	*t*	*p*-value	99% CI	*R* ^2^
PTSD symptoms	0.02	4.92	0.0002	0.011, 0.033	0.07	0.01	2.57	0.012	0.002, 0.018	0.13	−0.01	−3.30	0.001	−0.020, −0.005	0.10
Language	−0.14	−0.68	0.501	−0.565, 0.278		−0.14	−95	0.342	−0.434, 0.162		0.13	0.95	0.346	−0.148, 0.419	
PTSD symptoms x Language	−0.02	−2.45	0.016	−0.038, −0.004		−0.005	−0.83	0.409	−0.017, 0.007		0.01	2.02	0.046	0.0002, 0.023	

**Table 5:** Conditional effects language in the relationship between post-traumatic stress symptoms and distress and arousal.

Catastrophizing component-Dependent variable combination	Language	*β*	*t*	*p*-value	99% CI
PTSD symptoms-distress	Native	0.02	3.922	0.0002	0.011, 0.034
	Foreign	0.001	0.178	0.859	−0.012, 0.014
PTSD symptoms-arousal	Native	−0.01	−3.299	0.001	−0.020, −0.005
	Foreign	0.001	−0.197	0.844	−0.010, 0.008

## 4. Discussion

The present study is the first to explore whether the language of retrieval modulates the connection between post-traumatic stress symptoms and the level of emotionality related to the retrieval of a negative event. In particular, distress, valence and arousal when processing a negative autobiographical event.

As proposed in the initial hypothesis, the results showed that the relationship between the strength of traumatic symptoms and self-reported emotionality, specifically distress and arousal, is moderated by the language used. In particular, this strength is greater when the native language is used compared with a foreign language. These results are consistent with previous studies that support that using a native language produces a stronger emotional response than using a foreign language (Pavlenko, 2012; Iacozza et al., 2017; Dylman and Bjärtå, 2018). Although the causation of these results was not tested in this study, a plausible explanation for these results is the use of attentional resources. Specifically, attentional resources might be divided between using a language that is not mastered and the remembered event, resulting in less focus on the event itself and consequently working as a psychological or emotional barrier when managing the emotionality that the processing provokes. In this regard, neural studies have shown that grammatical and lexico-semantic processing have been designated as more cognitively demanding when a foreign language is used (Abutalebi, 2008).

Although the results of this study showed that people with more post-traumatic stress symptoms showed higher distress, arousal, and unpleasantness when recalling a negative event, the moderation effect of language was not found for the relationship between post-traumatic stress symptoms and valence ratings. These results suggest that the valence of a negative event changes after processing a negative event, but this effect is not influenced by the language used. Thus, language could have a greater impact on measures linked to physiological activation, such as arousal or distress-which is a common measure to assess fear activation during exposure procedures (e.g., van Deursen et al., 2021).

These results have important clinical implications for bilingual and multilingual patients. Our results show that emotional reactivity is forecasted by post-traumatic stress symptoms, being greater as those increase. As previously mentioned, some patients are reluctant to address their negative memories due to their high anxiety and stress when remembering them. Therefore, it seems feasible to use a foreign language when the post-traumatic stress symptoms are high to reduce the emotional reactivity whilst processing the negative experience. Hence, language may be an interesting tool when psychotherapists need to decrease emotionality to make a first approximation to traumatic experiences.

However, using a foreign language could also have some inconveniences in psychotherapy. Using a foreign language could entail a higher detachment during processing negative events, complicating some psychotherapeutic processes such as debriefing. In fact, literature commonly signalled more structured past memories and more detailed descriptions when retrieved in the native language (Javier and Marcos, 1989; Marian and Neisser, 2000). Also, a qualitative study showed that patients tend to switch from their foreign to their native language when they retrieve certain negative experiences associated with emotions such as anger (Santiago-Rivera et al., 2009). Therefore, language plays a significant role in the retrieving of memories, which should be used with caution by psychotherapists depending on the goal of the moment of the therapy. For example, language might be used as a first approach to negative autobiographical memories that patients consider challenging to retrieve or produce dissociative symptomatology. However, it would not be recommended in the latest steps of the exposition process since it could be used as a detachment strategy to reduce the emotionality produced by the memory.

This study has some strengths since it suggests that bilinguals with high post-traumatic stress symptomatology might benefit from a foreign language to reduce emotionality- in terms of distress, arousal and emotional valence- when retrieving a negative memory. These results open the door to a different approach for working with negative autobiographical memories, in which language plays a primary role during the psychotherapeutic process. These findings support the idea by Dewaele and Costa (2013) of the need to pay more attention to language as an influential factor within the multilingual clinical context. Patients can use bilingualism or multilingualism to adjust their needs for communication in therapy, as discussed by Dewaele et al. (2020). Even though we have not explained the phenomenon of the foreign language effect, we can bring some perspective to how it works in relation to negative memories and their processing, and ultimately the modulatory role this effect has on emotional response. Still, different paradigms and tasks remain to be explored, especially in relation to negatively charged events.

Even so, there are some limitations to consider in this study. One may be the recruitment of the sample. As this experiment explores a healthy population, the scores are concentrated in low scores in traumatic symptomatology, and the differences might be more evident when the DTS scores are higher. Therefore, conducting studies with clinical populations such as trauma or other disorders associated with stressful events (e.g., adaptive disorder) is recommended. Concerning language, it would be appropriate to specify whether language differences are due to the language itself or language switching, as, in this particular task, participants in the foreign language group had to switch from their native language to their foreign for a few items. Recent research has already pointed out the fact that switching from a native language to a foreign one might lead to facilitation in the processing of negative responses (Zang et al., 2022). Also, proficiency level in the foreign language should be measured objectively apart from subjectively, to obtain a more accurate measurement. In addition, although participants were asked to recall memories from childhood, some reported memories experienced in adulthood; this could imply different outcomes depending on the distance between the memory and the period in which it was experienced. Recent memories, for example, are typically perceived as more vivid and emotionally intense than remote memories (Sutin and Robins, 2007). In the same way, it would be recommendable that future studies also consider the age that has passed from the event that occurred since adults remember positive and negative information differently as time passes or after repeated exposure (Alves et al., 2017). Finally, further studies are needed to explore the foreign language effect in relation to different patient and event characteristics.

In conclusion, our results are consistent with prior results focused on studying the foreign language effect. Although we cannot confirm with these results that a different language directly evokes a different emotional intensity, these findings suggest that a foreign language can modulate the emotional expression of certain aspects of post-traumatic stress symptoms. These results are essential for psychotherapists, who may use language as a tool of detachment for patients with high post-traumatic stress symptomatology that are processing their negative experiences to reduce their emotional reactivity.

## Data availability statement

The datasets presented in this study can be found in online repositories. The names of the repository/repositories and accession number(s) can be found at: https://osf.io/n4wvk.

## Ethics statement

The study was reviewed and approved by University Jaume I (CD/100/2021). The participants provided their written informed consent to participate in this study.

## Author contributions

AG-P, IJ, and IO-B developed the study concept and provided ideas for the study design alongside IJ. IO-B prepared the questionnaires on the online platform. IO-B implemented the data collection and the testing of the participants. IJ executed the data analysis and interpretation of the results alongside IO-B. IO-B elaborated the manuscript with IJ under the supervision of AG-P. All authors contributed to the article and approved the submitted version.

## Funding

This work was supported by Marie Sklodowska-Curie Innovative Training Networks (ITN-ETN)—Multimind (H2020 MSCA-ITN-2017, grant 765556).

## Conflict of interest

The authors declare that the research was conducted in the absence of any commercial or financial relationships that could be construed as a potential conflict of interest.

## Publisher’s note

All claims expressed in this article are solely those of the authors and do not necessarily represent those of their affiliated organizations, or those of the publisher, the editors and the reviewers. Any product that may be evaluated in this article, or claim that may be made by its manufacturer, is not guaranteed or endorsed by the publisher.

## Footnotes

^1^ https://www.qualtrics.com

^2^ www.prolific.co
